# A Web-Based Integrated Management Program for Improving Medication Adherence and Quality of Life, and Reducing Readmission in Patients With Atrial Fibrillation: Randomized Controlled Trial

**DOI:** 10.2196/30107

**Published:** 2021-09-22

**Authors:** Hui-Ling Hsieh, Chi-Wen Kao, Shu-Meng Cheng, Yue-Cune Chang

**Affiliations:** 1 Graduate Institute of Medical Sciences National Defense Medical Center Taipei Taiwan; 2 School of Nursing National Defense Medical Center Taipei Taiwan; 3 Division of Cardiology, Department of Internal Medicine Tri-Service General Hospital, National Defense Medical Center Taipei Taiwan; 4 Department of Mathematics Tamkang University Taipei Taiwan

**Keywords:** web-based program, atrial fibrillation, coping strategy, medication adherence, readmission, health-related quality of life

## Abstract

**Background:**

Atrial fibrillation (AF) is related to a variety of chronic diseases and life-threatening complications. It is estimated that by 2050, there will be 72 million patients with AF in Asia, of which 2.9 million will have AF-associated stroke. AF has become a major issue for health care systems.

**Objective:**

We aimed to evaluate the effects of a web-based integrated management program on improving coping strategies, medication adherence, and health-related quality of life (HRQoL) in patients with AF, and to detect the effect on decreasing readmission events.

**Methods:**

The parallel-group, single-blind, prospective randomized controlled trial recruited patients with AF from a medical center in northern Taiwan and divided them randomly into intervention and control groups. Patients in the intervention group received the web-based integrated management program, whereas those in the control group received usual care. The measurement tools included the Brief Coping Orientation to Problems Experienced (COPE) scale, Medication Adherence Rating Scale (MARS), the three-level version of the EuroQoL five-dimension self-report questionnaire (EQ-5D-3L), and readmission events 2 years after initiating the intervention. Data were collected at 4 instances (baseline, 1 month, 3 months, and 6 months after initiating the intervention), and analyzed with generalized estimating equations (GEEs).

**Results:**

A total of 231 patients were recruited and allocated into an intervention (n=115) or control (n=116) group. The mean age of participants was 73.08 (SD 11.71) years. Most participants were diagnosed with paroxysmal AF (171/231, 74%), and the most frequent comorbidity was hypertension (162/231, 70.1%). Compared with the control group, the intervention group showed significantly greater improvement in approach coping strategies, medication adherence, and HRQoL at 1, 3, and 6 months (all *P*<.05). In addition, the intervention group showed significantly fewer readmission events within 2 years (OR 0.406, *P*=.03), compared with the control group.

**Conclusions:**

The web-based integrated management program can significantly improve patients' coping strategy and medication adherence. Therefore, it can empower patients to maintain disease stability, which is a major factor in improving their HRQoL and reducing readmission events within 2 years.

**Trial Registration:**

ClinicalTrials.gov NCT04813094; https://clinicaltrials.gov/ct2/show/NCT04813094.

## Introduction

Atrial fibrillation (AF) is related to a variety of chronic diseases and life-threatening complications [1]. Owing to progressive aging of the population and the force of unhealthy lifestyles, the global incidence of AF is expected to achieve a twofold increase in the next decade [2]. According to the 2010 Global Burden of Disease Study report, approximately 33 million people worldwide suffer from AF [3]. It is estimated that by 2050, there will be 72 million AF patients in Asia, of which 2.9 million will have AF-associated stroke [4]. Compared with Europe and the United States, the population of AF has increased more rapidly in Asia [5-7], suggesting that AF has become a major issue for Asian health care systems.

The asymptomatic nature of AF often makes patients unaware of the subsequent stroke [2]. Nearly 40% of AF patients suffer from cerebrovascular accidents for this reason [1,8,9]. AF is the most common cause of cardioembolic stroke, which is particularly severe in terms of high mortality and serious disability [2]. The complications of AF lead to frequent visits to the emergency department, hospitalization and readmission, and impaired quality of life [10-12].

Anticoagulation is the preferred medical intervention to prevent stroke in patients with AF [7]. Nonetheless, a European clinical trial showed that nearly 40% of patients with AF withdrew anticoagulants within 1 year following the initiation of the treatment [13]. In fact, compliance to anticoagulants decreases with time in patients with AF [14], which is a major factor contributing to stroke in this population [15,16]. As such, anticoagulant incompliance worsens the prognosis of AF in terms of increased readmission and mortality rates [17-19]. Ensuring anticoagulant adherence in patients with AF is an important issue for stroke prevention and disease recovery.

AF frequently occurs in patients with multiple concurrent chronic diseases including hypertension, ischemic heart disease, and heart failure, which increase the number of medications used. Patients taking several medications have many adverse effects and tend to be noncompliant to treatments [20]. Therefore, advanced and integrated disease management programs are essential for AF patients to achieve successful disease control [21]. Prior studies support the use of eHealth to assist patients in conducting advanced disease management. A few randomized controlled trials had investigated the impact of eHealth on medication adherence in patients with AF for 3 months [22,23]. Although the use of eHealth management significantly improved medication adherence at 3 months after intervention, the long-term effects on medication adherence remain to be investigated [24]. Again, integrated management aims to improve patients' disease knowledge and empower patients to manage their disease [9,24,25]. Integrated management needs the involvement of patients and multidisciplinary AF teams. Multidisciplinary professionals using technological support such as eHeath provide disease information and self-care skills to AF patients [9]. As a result, advanced and integrated disease management programs are expected to improve medication adherence and disease control in patients with AF.

In this study, we aimed to evaluate the effects of a web-based integrated management program on improving coping strategies, medication adherence, and health-related quality of life (HRQoL) in patients with AF, and to detect its effect on decreasing readmission events. Therefore, we have two hypotheses. The first hypothesis is that the AF patients receiving the web-based integrated management program in the intervention group would use more approach coping strategies and improve their medication adherence and HRQoL compared with the patients in the control group. The second hypothesis is that the patients in the intervention group would have fewer 2-year readmission events than the patients in the control group.

## Methods

### Design

This study was a prospective, single-blind, randomized controlled trial with repeated measurements to determine the effects of a 6-month web-based integrated management program on improving the coping strategy, medication adherence, and HRQoL, and decreasing 2-year readmission events in patients with AF. Data were collected from October 2018 to January 2021. We collected the data at 4 time points: baseline (prior to randomization, T0), 1 month after beginning the intervention (T1), after completing 3 months of the intervention (T2), and after completing 6 months of the intervention (T3).

### Participants

Participants were recruited from the cardiovascular outpatient department at a medical center in northern Taiwan through convenience sampling. Patients were included if they met the following criteria: (1) diagnosed with AF by cardiologists, (2) receiving anticoagulant treatment, (3) aged above 20 years, (4) able to speak and read Taiwanese or Mandarin to understand and follow instructions, and (5) able to use a mobile phone or computer correctly. The exclusion criteria included the following: (1) diagnosed with mental disorders or (2) involved in other clinical trials.

In total, 324 patients were screened in the cardiovascular outpatient department, and 258 patients were eligible to be included. Among the eligible patients, 26 patients refused to participate. The remaining 232 participants were randomly assigned into 2 groups by blocked randomization using a web-based system [26]. Finally, 116 participants were assigned into the intervention group and 116 participants were assigned into the control group. The patients in the intervention group received the web-based integrated management program, whereas those in the control group received consultations and were coached thrice by a research nurse over telephone.

### Ethical Considerations

This study was approved by the Institutional Review Board of the participating hospital (IRB: 2-107-05-081; ClinicalTrials.gov NCT04813094) and conducted in accordance with the principles of the Declaration of Helsinki revised in 2000. A research nurse explained the research process to each participant and obtained informed consent from them. We ensured that the participants were kept anonymous. In addition, participants were informed that they had the right to withdraw from the study at any time for any reason.

### Study Interventions

**Figure 1:**
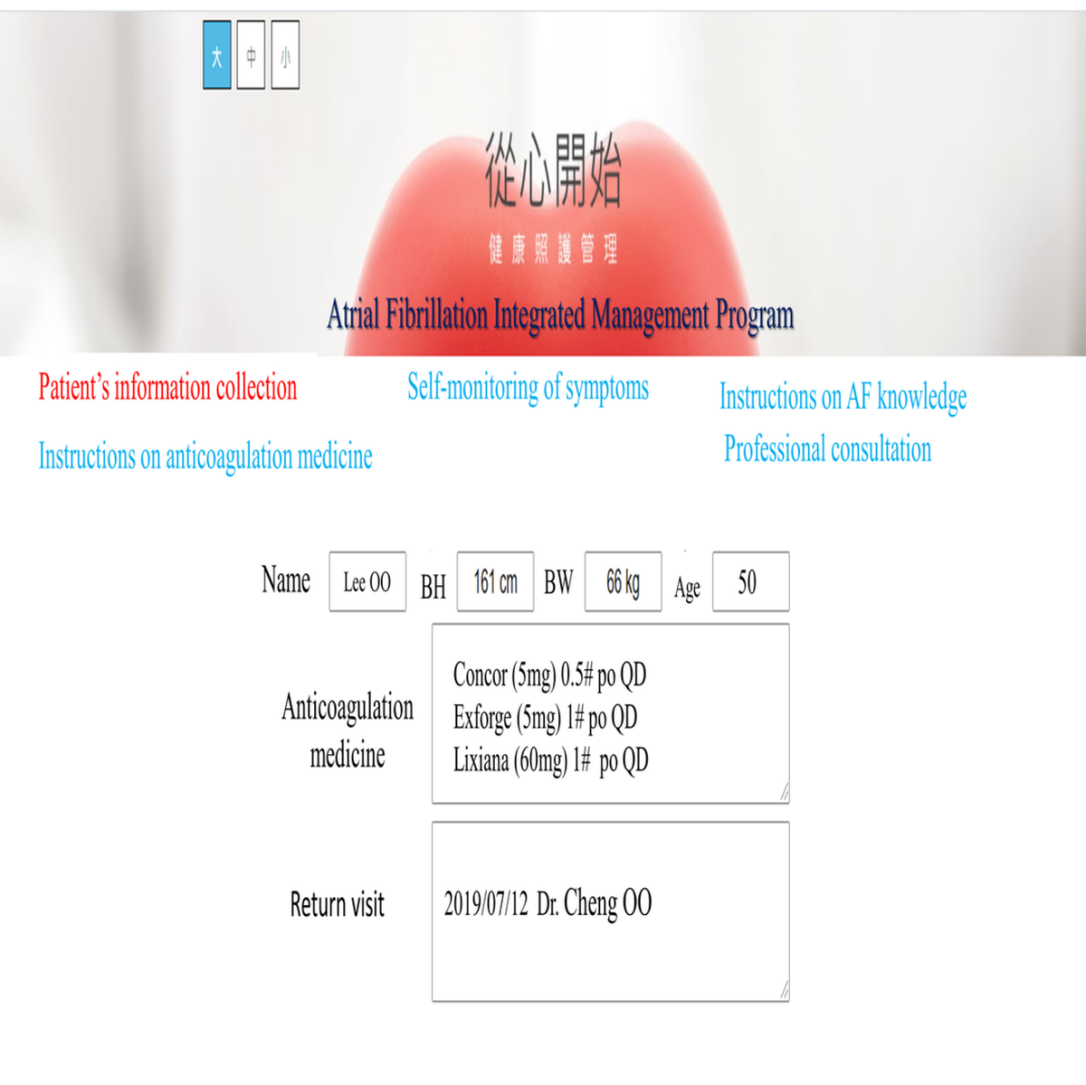
Screenshot of the Atrial Fibrillation Integrated Management Program. BH: body height; BW: body weight; po: per os (Latin term meaning orally) QD: qaque die (Latin term meaning one a day).

**Figure 2:**
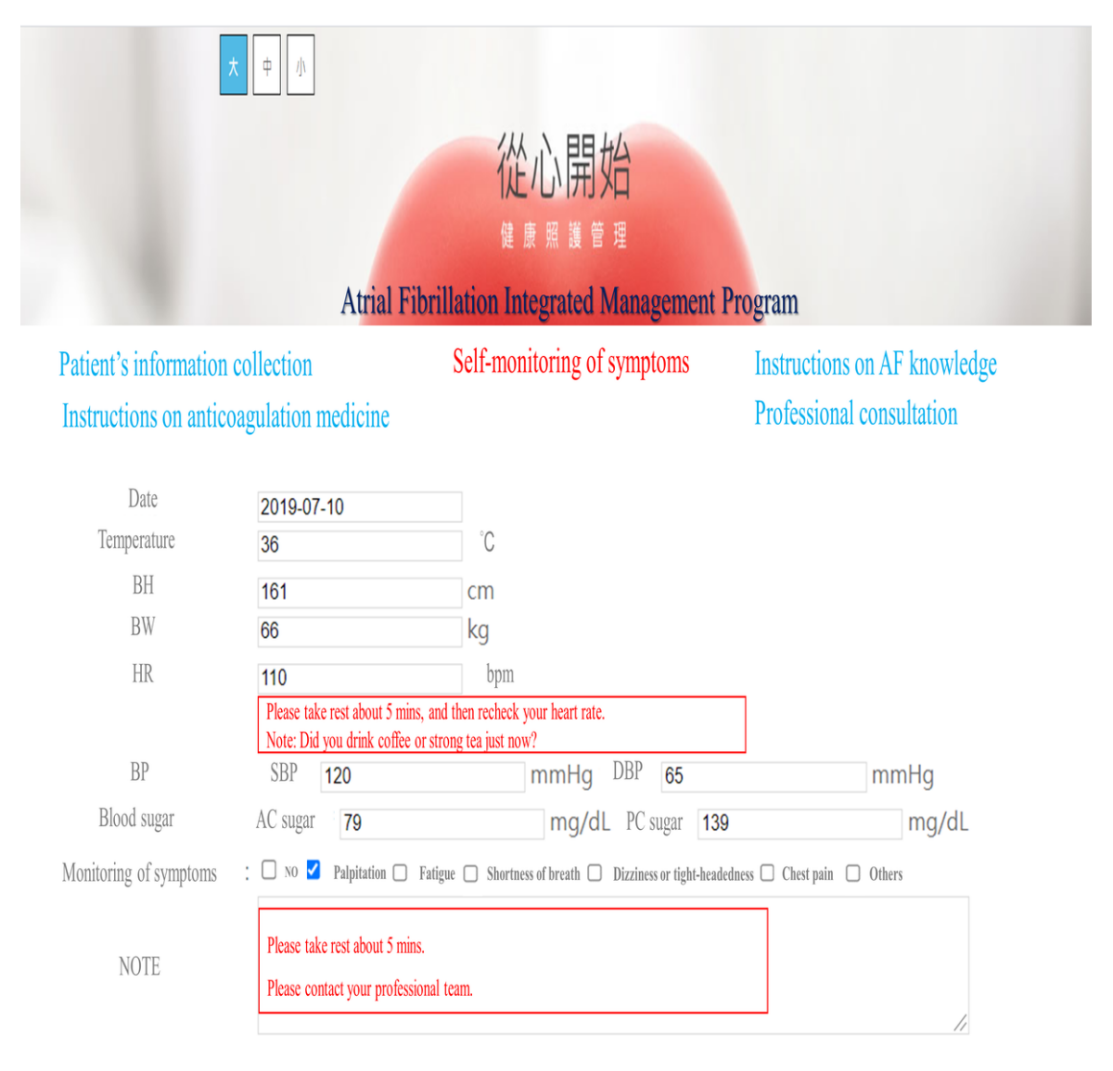
Screenshot showing self-monitoring of symptoms. AF: atrial fibrillation; BH: body height; BW: body weight; HR: heart rate; BP: blood pressure; DBP: diastolic blood pressure; SBP: systolic blood pressure.

The web-based integrated management program was conducted in the cardiovascular outpatient department. Participants had their own account and password to log in to the web-based program via mobile phones or computers. The program included five domains: patient information collection, instructions on AF knowledge, instructions on anticoagulation medicine, self-monitoring of symptoms, and professional consultation. In the patient information collection domain, the participants were able to provide and read their information, including age, gender, years of education, type of AF, modified European Heart Rhythm Association (mEHRA) classification, comorbidities, and use of anticoagulation medication. In the instructions on AF knowledge domain, participants were able to receive information about AF through texts and videos, including an introduction to AF, the risk factors and symptoms of AF, how to prevent stroke in patients with AF, and how to manage a healthy lifestyle. The videos were also available in Taiwanese and Mandarin. In the instructions on anticoagulation medicine domain, the participants were able to obtain descriptions about a variety of anticoagulant medicines. There was textual information about anticoagulant medicines, such as the effectiveness and adverse effects of these medicines, and precautions to be taken, as well as pictures of the corresponding medicines to serve as references for the participants. In the self-monitoring of symptoms domain, participants could provide record their symptoms every day, which could assist them in monitoring their disease progress and provide cardiologists with references to regulate their treatment. In the professional consultation domain, participants could receive consultations from multidisciplinary professionals on any issues related to AF at any time. All the participants had their own private consultations with clinical professionals. The research nurse also sent messages every day to monitor the participants' condition through the messaging function of this domain. When the participants had an emergency event, they could receive help to manage this situation through textual information or telephonic coaching (Figures 1 and 2).

After face-to-face presentations, we ensured that the participants fully understood the instructions for using this web-based program by the return demonstration method. We reviewed their understanding of the program at the next outpatient visit. In addition, we instructed participants on managing AF and anticoagulant treatment through the web-based program and provided the AF management manual to them.

### Control Group

Patients in the control group received standard instructions. The AF management manual was provided to them with explanations. In addition, we provided telephonic coaching thrice, which taught participants how to manage their disease at 1 month, 3 months, and 6 months after random assignment.

### Measurements

#### The Clinical and Demographic Information Form

Demographic and clinical characteristics, including age, gender, education, marital status, type of AF, mEHRA classification, comorbidities, and use of anticoagulation medicine, were recorded before random assignment. The mEHRA classification was used to categorize AF patients according to their symptom severity. The mEHRA classification has five levels including class I, none; class IIa, mild; class IIb, moderate; class III, severe; and class IV, disabling [9].

#### Coping Strategies

The participants’ coping strategies were measured using the Brief Coping Orientation to Problems Experienced (COPE) scale [27]. This 28-item instrument contains 2 subscales: approach coping (8 items) and avoidance coping (6 items). The approach coping strategies show how individuals actively seek resources to deal with their health problems (active coping, planning, positive reframing, acceptance, humor, religion, and using instrumental and emotional support). The avoidance coping strategies show how individuals attempt to divert attention away from events (self-distraction, denial, venting, substance use, behavioral disengagement, and self-blame) [28]. A higher score indicated that the patient often uses this coping strategy. In this study, the Brief COPE was translated into Chinese. The content validity for the Chinese version of the Brief COPE was examined by five cardiology experts. The content validity index was 0.83. A previous study reported that the Cronbach α of each item ranged between .50 and .90 [27].

#### Medication Adherence

The participants’ medication adherence was measured by the Medication Adherence Rating Scale (MARS) [29]. This instrument includes three domains: medication adherence behavior, attitude toward taking medication, and attitude toward the adverse effects of medication. Each item has two options. Participants choose an answer based on their prescription. The total possible score ranges from 0 to 10. A higher score indicates better medication adherence. In this study, the MARS was translated into its Chinese version. The content validity of the Chinese version was examined by five cardiology experts. The content validity index was 0.92. The Cronbach α of the MARS was .75 [29].

#### HRQoL Measurement

The participants’ HRQoL was measured by the three-level version of the EuroQol five-dimension self-report questionnaire (EQ-5D-3L). The instrument includes 2 subscales: the EQ-5D descriptive system (5 items) and EuroQol visual analog scale (EQ-VAS). The EQ-5D descriptive system had five domains: mobility, self-care, usual activities, pain/discomfort, and anxiety/depression. Each item was rated at 3 levels: no problems at level 1 and extreme problems at level 3. The scores were converted into a single summary score. The EQ-VAS was a 20 cm visual analog scale to let participants self-assess their health status. The range of the EQ-VAS was from 0 to 100. A high score indicated the best state of health. We used the Chinese version of the EQ-5D-3L, which has adequate validity and reliability [30].

#### Readmission Events Within 2 Years

After recruiting the patients in the study, we followed their readmission events for 2 years. We collected these data through chart reviews of each participant.

#### Data Analysis

Statistical analyses were conducted using SPSS (version 23.0; IBM Corp). Categorical variables were reported as frequencies and percentages, and continuous variables with normal distributions were reported as means with SDs. The initial differences between groups for demographic characteristics and the baseline scores of each scale were examined with independent *t* and chi-square tests. The effects of the web-based integrated management program on the coping strategy, medication adherence, HRQoL, and readmission events were examined using a generalized estimating equation (GEE) [31]. The significance level was defined by a two-tailed *t* test with *P*<.05.

We used univariate logistic regression to assess the association between patient characteristics, including group assignment, and readmission events within 2 years and identify the predictor variables. The readmission event within 2 years was coded as the dependent variable with 1 denoting a readmission event within 2 years after intervention and 0 denoting no readmission event within 2 years after intervention. The predictor variables with *P*<.20 were eligible for inclusion in the multivariable logistic regression model for measuring the outcome. In the multivariable logistic regression model, predictor variables with two-tailed *t* tests and *P*<.05 were considered significant [32]. We used the GPower (Version 3.1, Heinrich-Heine-Universität [33]) procedure with an α value of .05, a medium effect size of 0.25, and sample size of 231 to conduct a post hoc statistical power analysis [33]. Finally, the statistical power of this study was greater than 0.8.

## Results

### Participant Characteristics

**Figure 3:**
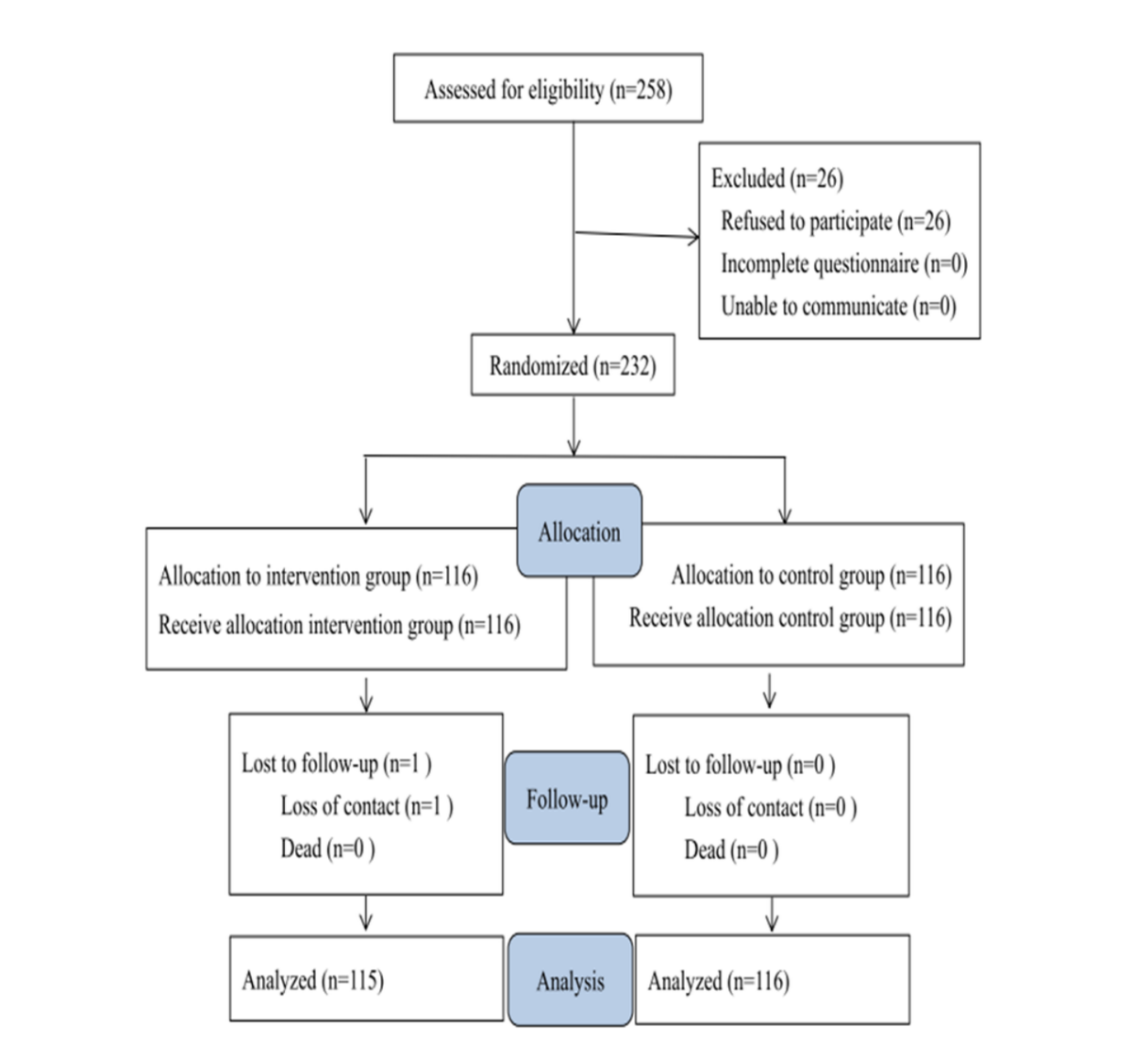
Flow diagram showing the inclusion of patients with atrial fibrillation in the two groups for the randomized control trial.

A total of 258 patients were recruited from the outpatient department of the study hospital according to the inclusion criteria. Of these patients, 26 refused to participate. At the end of the study, the complete data of 115 participants in the intervention group and 116 participants in the control group were included for the statistical analysis. One participant in the intervention group was lost to follow-up owing to emigration (Figure 3). The mean age of the participants was 73.08 (SD 11.71) years. Half of the participants were male (116/231, 50.2%), and over half of the participants were married (155/231, 67.1%). Most participants were diagnosed with paroxysmal AF (171/231, 74%), and the most frequent comorbidity was hypertension (162/231, 70.1%). Assessing the classification of AF symptoms through mEHRA indicated that 44.6% (103/231) of the participants were class I, 22.9% (53/231) were class IIa, 14.7% (34/231) were class IIb, and 17.7% (41/231) were class III. Approximately 36.4% (84/231) of the participants received rivaroxaban, and 27.3% (63/231) received apixaban. There were no significant differences in the demographic and clinical characteristics, and the baseline scores of each scale between the two groups (Table 1).

**Table 1:** Baseline characteristics of the study participants (N=231) and differences between groups.

Characteristic			Total (N=231)	Intervention group (n=115)	Control group (n=116)	*P* value
Age (years), mean (SD)			73.08 (11.71)	71.57 (13.16)	74.57 (9.9)	.052
**Gender, n (%)**						.19
	Male		116 (50.2)	63 (54.8)	53 (45.7)	
	Female		115 (49.8)	52 (45.2)	63 (54.3)	
**Education (years), n (%)**						.19
	<6 years		98 (42.4)	42 (36.5)	56 (48.3)	
	6-12 years		84 (36.4)	46 (40)	38 (32.8)	
	>12 years		49 (21.2)	27 (23.5)	22 (19)	
**Marital status, n (%)**						.75
	Single		6 (2.6)	4 (3.5)	2 (1.7)	
	Married		155 (67.1)	78 (67.8)	77 (66.4)	
	Divorced		7 (3)	4 (3.5)	3 (2.6)	
	Widowed		63(27.3)	29 (25.2)	34 (29.3)	
**Type of AF,^a^ n (%)**						.17
	Paroxysmal AF		171 (74)	85 (73.9)	86 (74.1)	
	Persistent AF		21 (9.1)	14 (12.2)	7 (6)	
	Permanent AF		39 (16.9)	16 (13.9)	23 (19.8)	
**mEHRA,^b^ n (%)**						.88
	mEHRA I		103 (44.6)	52 (45.2)	51 (44)	
	mEHRA IIa		53 (22.9)	28 (23.4)	25 (21.6)	
	mEHRA IIb		34 (14.7)	15 (13)	19 (16.4)	
	mEHRA III		41 (17.7)	20 (17.4)	21 (18.1)	
	mEHRA IV		0 (0)	0 (0)	0 (0)	
**Comorbidities, n (%)**						
	Hypertension		162 (70.1)	77 (67)	85 (73.3)	.32
	Diabetes mellitus		61 (26.4)	26 (22.6)	35 (30.2)	.23
	MI^c^		14 (6.1)	7 (6.1)	7 (6)	.99
	Valve heart disease		77 (33.3)	36 (31.3)	41 (35.3)	.58
	Valve heart disease		77 (33.3)	36 (31.3)	41 (35.3)	.58
	Heart failure		78 (33.8)	39 (33.9)	39 (33.6)	.99
	CAD^d^		85 (36.8)	41 (35.7)	44 (37.9)	.79
	Hyperthyroid		14 (6.1)	5 (4.3)	9 (7.8)	.41
	Cancer		10 (4.3)	5 (4.3)	5 (4.3)	.99
**Medication, n (%)**						
	Ticlopidine hydrochloride		1 (0.4)	1 (0.9)	0 (0)	.50
	Dipyridamole		6 (2.6)	4 (3.5)	2 (1.7)	.45
	Aspirin		17 (7.4)	10 (8.7)	7 (6)	.46
	Apixaban		63 (27.3)	32 (27.8)	31 (26.7)	.88
	Edoxaban		47 (20.3)	28 (24.3)	19 (16.4)	.14
	Clopidogrel		10 (4.3)	3 (2.6)	7 (6)	.33
	Dabigatran		7 (3)	3 (2.6)	4 (3.4)	.99
	Rivaroxaban		84 (36.4)	38 (33)	46 (39.7)	.34
**Questionnaires, mean (SD)**						
	**Brief COPE^e^**					
		Approach coping	26.1 (7.68)	25.6 (7.58)	26.6 (7.77)	.33
		Avoidance coping	14.32 (6.04)	15.02 (6.6)	13.63 (5.36)	.08
	MARS^f^		7.07 (1.80)	7.17 (1.79)	6.97 (1.80)	.40
	HRQoL^g^					
EQ-5D^h^			0.62 (0.12)	0.61 (0.12)	0.63 (0.12)	.16
EQ-VAS^i^			81.22 (6.48)	80.57 (7.079)	81.85 (5.79)	.13

^a^AF: atrial fibrillation.

^b^mEHRA: modified European Heart Rhythm Association.

^c^MI: myocardial infarction.

^d^CAD: coronary arterial disease.

^e^Brief COPE: Brief Coping Orientation to Problems Experienced.

^f^MARS: Medication Adherence Rating Scale.

^g^HRQoL: health-related quality of life.

^h^EQ-5D: EuroQol five-dimension self-report questionnaire.

^i^EQ-VAS: EuroQol visual analog scale.

### Effects on Coping Strategies

**Figure 4:**
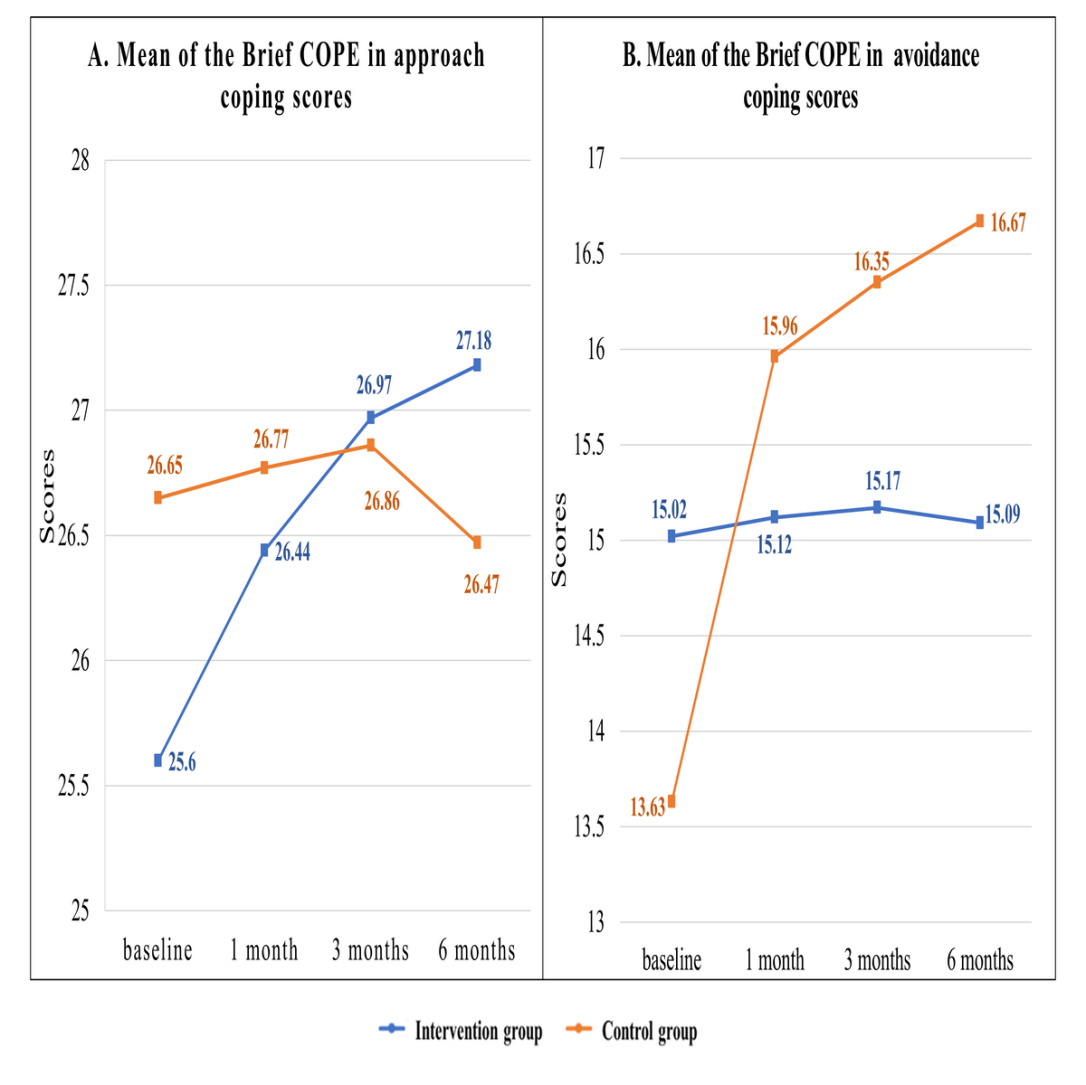
Mean scores of the approach and avoidance coping subscales for participants in the intervention and control groups. Brief COPE: Brief Coping Orientation to Problems Experienced.

The GEE model analysis showed a significant difference in the scores of the approach coping subscale between the intervention and control groups at 1 month (β=.792; 95% CI 0.143-1.172; *P*<.001), 3-months (β=1.297; 95% CI 0.441-2.153; *P*=.003), and 6 months (β=1.902; 95% CI 0.882-2.922; *P<*.001) (Table 2 and Figure 4).

In addition, the intervention group showed a significantly higher decrease in the scores of the avoidance coping subscale at 1 month (β=–2.284; 95% CI –2.885 to –1.683; *P*<.001), 3 months (β=–2.602; 95% CI –3.511 to –1.694; *P*<.001), and 6 months (β=–2.982; 95% CI –4.096 to –1.869; *P*<.001) compared with the control group (Table 2 and Figure 4). These findings indicated that the web-based integrated management program helped participants to use more approach coping strategies and less avoidance coping strategies in managing their AF-related issues.

**Table 2:** Generalized estimating equation analysis of the intervention effect on the Brief Coping Orientation to Problems Experienced scale (N=231).

Variable			β	95% CI	SE^a^	χ^2^	*P* value
**Approach coping**							
	Group (intervention)^b^		–.995	–2.966 to 0.976	1.006	0.979	.32
	**Time** **effects**						
		Time (1-month, T1)^c^	.129	–0.070 to –0.329	0.102	1.618	.20
		Time (3-months, T2)	.155	–0.479 to 0.789	0.323	0.230	.63
		Time (6-months, T3)^c^	–.241	–0.989 to 0.506	0.381	0.401	.53
	**Interaction effects**						
		Intervention × T1^d^	.792	0.143 to 1.172	0.194	16.778	<.001
		Intervention × T2^d^	1.297	0.441 to 2.153	0.437	8.816	.003
		Intervention × T3^d^	1.902	0.882 to 2.922	0.520	13.362	<.001
**Avoidance coping**							
	Group (intervention)^b^		1.388	–0.157 to 2.933	0.788	3.102	.078
	**Time effects**						
		Time (1 month, T1)^c^	2.388	1.856 to 2.920	0.272	77.383	<.001
		Time (3 months, T2)	2.724	1.966 to 3.483	0.387	49.552	<.001
		Time (6 months, T3)^c^	3.043	2.240 to 3.846	0.410	55.215	<.001
	**Interaction effects**						
		Intervention × T1^d^	–2.284	–2.885 to –1.683	0.307	55.471	<.001
		Intervention × T2^d^	–2.602	–3.511 to –1.694	0.464	31.505	<.001
		Intervention × T3^d^	–2.982	–4.096 to –1.869	0.568	27.554	<.001

^a^SE: standard error.

^b^Reference group: control group.

^c^Reference group: time (baseline).

^d^Reference group: group (control) × time (baseline).

### Effects on Medication Adherence

**Figure 5:**
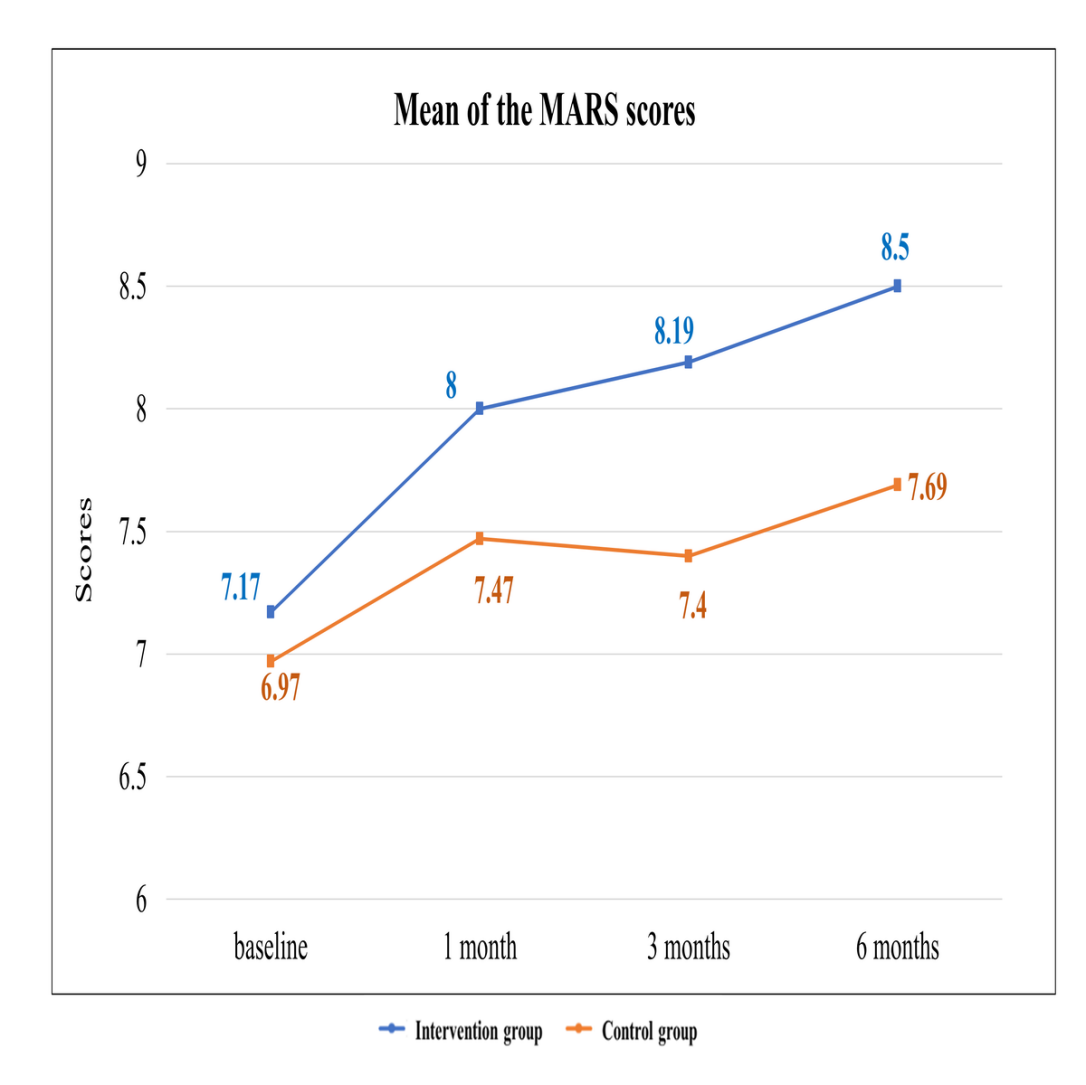
Mean scores of the Medication Adherence Rating Scale for participants in the intervention and control groups. MARS: Medication Adherence Rating Scale.

The GEE model analysis showed significant differences in the scores of the MARS between the intervention and control groups at 1 month (β=.326; 95% CI 0.034-0.618; *P*=.03), 3 months (β=.595; 95% CI 0.24-0.95; *P*=.001), and 6 months (β=.606; 95% CI 0.253-0.96; *P*=.001) (Table 3 and Figure 5). These results indicated that after receiving the web-based integrated management program, the AF patients showed significantly better medication adherence than that shown by the patients in the control group.

**Table 3:** Generalized estimation equation analysis of the intervention effect on Medication Adherence Rating Scale (N=231).

Variable		β	95% CI	SE^a^	χ^2^	*P* value
Group (intervention)^b^		.2	–0.262 to 0.661	0.2354	0.720	.40
**Time effects**						
	Time (1-month, T1)^c^	.5	0.337 to 0.663	0.831	36.172	<.001
	Time (3-months, T2)	.422	0.181 to 0.664	0.1232	11.752	.001
	Time (6-months, T3)^c^	.716	0.499 to 0.932	0.1103	42.106	<.001
**Interaction effects**						
	Intervention × T1^d^	.326	0.034 to 0.618	0.149	4.784	.03
	Intervention × T2^d^	.595	0.240 to 0.950	0.181	10.818	.001
	Intervention × T3^d^	.606	0.253 to 0.960	0.180	11.306	.001

^a^SE: standard error.

^b^Reference group: control group.

^c^Reference group: time (baseline).

^d^Reference group: group (control) × time (baseline).

### Effects on HRQoL

**Figure 6:**
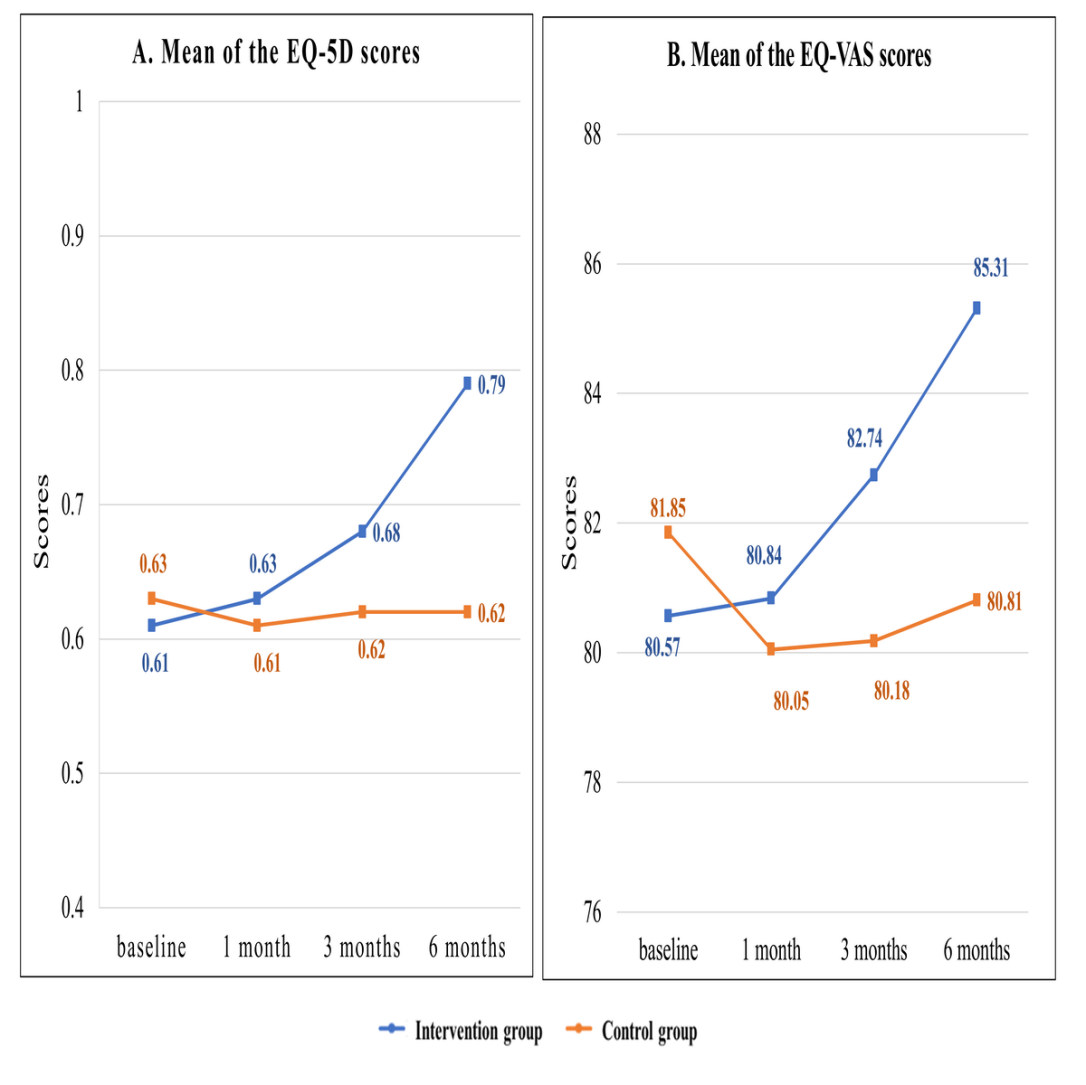
Mean scores of the EuroQoL five-dimension self-report questionnaire and EuroQoL visual analog scale for participants in the intervention and control groups. EQ-5D: EuroQoL five-dimension self-report questionnaire; EQ-VAS: EuroQoL visual analog scale.

The GEE model analysis showed no significant difference in the EQ-5D scores between the 2 groups at 1 month (β=.042; 95% CI –0.014 to 0.980; *P=*.14). However, the patients in the intervention group showed significantly higher EQ-5D scores at 3 months (β=.082; 95% CI 0.028-0.137; *P*=.003) and 6 months (β=.19; 95% CI 0.13-0.25; *P*<.001) than the patients in the control group (Table 4 and Figure 6).

In addition, the GEE model analysis revealed significant differences between the 2 groups in the EQ-VAS scores at 1 month (β=2.071; 95% CI 0.551-3.592; *P=*.008), 3 months (β=3.838; 95% CI 2.169-5.506; *P<*.001), and 6 months (β=5.782; 95% CI 3.837-7.727; *P<*.001) (Table 4 and Figure 6). These results indicated that the AF patients receiving the web-based integrated management program showed a significantly higher HRQoL improvement than that shown by the patients in the control group.

**Table 4:** Generalized estimating equation analysis of the intervention effect on health-related quality of life (N=231).

Variable		β	95% CI	SE^a^	χ^2^	*P* value
**EQ-5D score^b^**						
	Group (intervention)^c^	–.022	–0.053 to 0.010	0.016	1.717	.16
**Time effects**						
	Time (1-months, T1)^d^	–.020	–0.055 to 0.015	0.018	1.255	.26
	Time (3-months, T2)^d^	–.012	–0.041 to 0.017	0.015	0.642	.42
	Time (6-months, T3)^d^	–.010	–0.059 to 0.040	0.025	0.153	.70
**Interaction effects**						
	Intervention × T1^e^	.042	–0.014 to 0.098	0.285	2.168	.14
	Intervention × T2^e^	.082	0.028 to 0.137	0.028	8.820	.003
	Intervention × T3^e^	.190	0.130 to 0.250	0.031	38.222	<.001
**EQ-VAS^f^ score**						
	Group (intervention)^c^	–1.280	–2.941 to 0.382	0.848	2.278	.13
**Time effects**						
	Time (1-months, T1)^d^	–1.802	–2.918 to –0.685	0.570	10.008	.002
	Time (3-months, T2)^d^	–1.672	–2.800 to –0.545	0.575	8.447	.004
	Time (6-months, T3)^d^	–1.043	–2.353 to 0.267	0.668	2.436	.12
**Interaction effects**						
	Intervention × T1^e^	2.071	0.551 to 3.592	0.776	7.128	.008
	Intervention × T2^e^	3.838	2.169 to 5.506	0.851	20.316	<.001
	Intervention × T3^e^	5.782	3.837 to 7.727	0.992	33.946	<.001

^a^SE: standard error.

^b^EQ-5D: EuroQol five-dimension self-report questionnaire.

^c^Reference group: control group.

^d^Reference group: time (baseline).

^e^Reference group: group (control) × time (baseline).

^f^EQ-VAS: EuroQol visual analog scale.

### Effects on Readmission Events Within 2 Years

During the study, we followed the readmission events within 2 years after intervention. A total of 34 readmission events occurred. There were 11 readmission events in the intervention group and 23 in the control group. The predictor variables with *P*<.20 were based on univariate logistic regression. The predictor variables meeting the criteria were patients aged over 65 years, the mEHRA IIa class, comorbidities such as valvular heart disease and heart failure, receiving clopidogrel, and group assignment (Table 5). According to the multivariate logistic regression model, the intervention group showed significantly fewer readmission events within 2 years (OR 0.406; 95% CI 0.178-0.926; *P=*.03) compared with the control group (Table 5). These results indicated that the patients with AF receiving the web-based integrated management program had a lower probability of readmission within 2 years, compared with the control group.

**Table 5:** Association between the web-based integrated management program and readmission events within 2 years (N=231).

Characteristic		Univariate			Multivariate		
		OR^a^ (%)	95% CI	*P* value	OR (%)	95% CI	*P* value
Gender: male		1.136	0.548-2.356	.73	—^b^	—	—
Aged over 65 years		0.574	0.253-1.300	.18	0.608	0.240 to –1.542	.30
**Type of AF^c^**							
	Persistent AF	0.929	0.255-3.382	.91	—	—	—
	Permanent AF	0.820	0.294-2.291	.71	—	—	—
**mEHRA^d^**							
	mEHRA IIa	0.444	0.141-1.402	.17	0.378	0.112-1.269	.16
	mEHRA IIb	0.725	0.225-2.340	.59	—	—	—
	mEHRA III	1.754	0.720-4.272	.22	—	—	—
**Comorbidities**							
	Hypertension	1.026	0.462-2.280	.95	—	—	—
	Diabetes mellitus	1.004	0.440-2.291	.99	—	—	—
	MI^e^	0.964	0.206-4.509	.96	—	—	—
	Valvular heart disease	1.982	0.947-4.416	.07	2.200	0.994-4.870	.05
	Heart failure	2.230	1.067-4.659	.03	1.907	0.851-4.272	.12
	CAD^f^	1.075	0.508-2.275	.85	—	—	—
	Hyperthyroidism	1.038	0.222-4.857	.96	—	—	—
	Cancer	1.477	0.300-7.271	.63	—	—	—
	Dyslipidemia	1.123	0.530-2.379	.76	—	—	—
**Medication**							
	Ticlopidine hydrochloride	0	—	.99	—	—	—
	Dipyridamole	1.164	0.132-10.279	.89	—	—	—
	Aspirin	0.343	0.044-2.674	.31	—	—	—
	Apixaban	1.132	0.508-2.525	.76	—	—	—
	Edoxaban	1.247	0.524-2.965	.62	—	—	—
	Clopidogrel	4.244	1.131-15.927	.03	4.240	0.843-21.324	.08
	Dabigatran	0	—	.99	—	—	—
	Rivaroxaban	0.693	0.314-1.529	.36	—	—	—
Intervention group^g^		0.428	0.198-0.925	.03	0.406	0.178-0.926	.03

^a^OR: odds ratio.

^b^Not applicable.

^c^AF: atrial fibrillation. Reference group: Paroxysmal AF.

^d^mEHRA: modified European Heart Rhythm Association. Reference group: mEHRA I.

^e^MI: myocardial infarction.

^f^CAD: coronary arterial disease.

^g^Reference group: control group.

## Discussion

### Principal Findings

The main findings of this study were that the web-based integrated management program significantly improved coping strategies, medication adherence, and HRQoL in patients with AF after 1, 3, and 6 months of intervention. In addition, the AF patients receiving the web-based integrated management program had a lower probability of readmission within 2 years after intervention compared with the patients not receiving this program.

The findings of this study identified that the AF patients receiving this program increased the usage of approach coping strategies and decreased the usage of avoidance coping strategies, as opposed to the patients in the control group. Individuals who used approach coping strategies were able to actively solve problems and conduct positive reappraisals [34]. As the patients were able to receive information immediately and appropriately through this web-based program, they became more confident in solving their problems. On the other hand, the patients in the control group received the manual for AF management to address their problems. They always complained that they did not have sufficient disease information and immediate discussions with clinical professionals when they faced problems. They resorted to using more avoidance coping strategies. Although avoidance coping strategies may be useful in temporarily reducing stress, they increase suffering and mortality, and decrease medication adherence and quality of life [35-37].

Furthermore, in our study, patients receiving the web-based program showed significant improvements in medication adherence and HRQoL, which resulted from the improved disease knowledge and empowerment to solve their problems. Wang et al pointed out three reasons why knowledge may improve medication adherence in patients with AF; first, they become more knowledgeable about the importance of anticoagulant treatment; second, they develop the ability to detect the adverse effects of anticoagulant medicines; third, they received more self-care information about managing the symptoms of AF and adverse effects of the medicines [38]. In addition, Ammenwerth et al suggested that integrated management with eHealth schemes can increase motivated lifestyles and adherence to medical recommendations, thereby improving the health status and HRQoL [39]. Our findings were consistent with those of previous studies [40,41]. All the findings proved that patients were able to manage their treatment and lifestyle by themselves through a web-based program providing positive coping strategies and sufficient disease knowledge and could therefore improve their medication adherence and HRQoL.

To test the second hypothesis that the patients in the intervention group would experience fewer 2-year readmission events than those in the control group, we followed the readmission status of AF patients for 2 years. Our findings showed significantly fewer readmission events within 2 years in patients who received the web-based integrated management program compared to those in the control group. Marcolino et al proposed that implementing web-based intervention programs can improve communication between patients and medical providers and provide assistance in disease management to promote health recovery of patients [42]. In our study, through the web-based integrated management program, direct communication between the patients/their families and health professionals was possible when the patients went back home. A randomized control trial involving patients with heart failure showed that using a remote medication monitoring system can greatly improve patients’ medication adherence and reduce all-cause readmission events. It reduced the risk of readmission by 80% [43]. Our findings were consistent with these previous ones [44,45]. Because of the web-based integrated management program, we empowered patients to change their coping strategies and increase their compliance to medical treatment. Ultimately, it improved the HRQoL and long-term beneficial clinical effects in patients with AF in terms of reducing 2-year readmission events.

### Conclusions

The study provides useful information for health care professionals regarding web-based integrated management programs for optimizing AF management. The most important findings of the study are summarized as follows: The web-based integrated management program can significantly improve patients' coping strategies and medication adherence, and help patients maintain disease stability, which has a major influence on improving the HRQoL and reducing adverse clinical events and readmission episodes. Overall, the program may improve the quality of patient care while reducing medical costs.

### Limitations

Some limitations of this study should be considered. First, the sample may not reflect the entire AF population owing to the disease severity of most patients being mild or moderate. If AF patients had severe symptoms, they were hospitalized to receive medical interventions. Therefore, most of the AF patients in the community only had mild or moderate symptoms. However, they still had very limited knowledge on self-managing their symptoms. After receiving this program, they were able to take care of themselves, leading to decreased readmission events. Second, the single-center design limited the generalization of the findings. Third, factors that affect medication adherence and readmission, such as social support and economic status, were not included in the present study. As there were no differences in the educational levels and marital statuses of the patients in the intervention and control groups, we assumed that both groups of patients had similar social support levels and economic statuses. Nevertheless, future studies should examine these two factors and control their influence.

## Appendix

Multimedia Appendix 1 CONSORT-eHEALTH checklist (V 1.6.2).

## Abbreviations

AF: atrial fibrillation
EQ-5D-3L: three-level version of EuroQol five-dimension self-report questionnaire
EQ-VAS: EuroQol visual analog scale
GEE: generalized estimating equation
HRQoL: health-related quality of life
MARS: Medication Adherence Rating Scale
mEHRA: modified European Heart Rhythm Association
