# Supplementary material for: A Web-Based Integrated Management Program for Improving Medication Adherence and Quality of Life, and Reducing Readmission in Patients With Atrial Fibrillation: Randomized Controlled Trial
Source: J Med Internet Res. 2021 Sep 22;23(9):e30107. doi: 10.2196/30107 (PMC8495568; doi:10.2196/30107)
Supplement: Multimedia Appendix 1 [file jmir_v23i9e30107_app1.pdf]

CONSORT-EHEALTH Checklist V1.6.2 Report	Manuscript Number	30107
**(based on CONSORT-EHEALTH V1.6), available at [http://tinyurl.com/consort-ehealth-v1-6].**		
**Date completed**		
8/31/2021 2:47:05		
**by**		
Chi-Wen Kao		
A Web-Based Integrated Management Program for Improving Medication Adherence and Quality of Life, and Reducing Readmission in Patients With Atrial Fibrillation: Randomized Controlled Trial		
TITLE		
**1a-i) Identify the mode of delivery in the title**		
"Randomized Controlled Trial"		
**1a-ii) Non-web-based components or important co-interventions in title**		
**1a-iii) Primary condition or target group in the title**		
..... "on improving coping strategies, medication adherence, and health-related quality of life (HRQoL) in patients with AF".		
ABSTRACT		
**1b-i) Key features/functionalities/components of the intervention and comparator in the METHODS section of the ABSTRACT**		
"This study was a prospective, single-blind, randomized controlled trial with repeated measurements to determine the effects of a 6-month web-based integrated management program on improving the coping strategy, medication adherence, and HRQoL, and decreasing 2-year readmission events in patients with AF".		
**1b-ii) Level of human involvement in the METHODS section of the ABSTRACT**		
**1b-iii) Open vs. closed, web-based (self-assessment) vs. face-to-face assessments in the METHODS section of the ABSTRACT**		
**1b-iv) RESULTS section in abstract must contain use data**		
**1b-v) CONCLUSIONS/DISCUSSION in abstract for negative trials**		
INTRODUCTION		
**2a-i) Problem and the type of system/solution**		
"The main findings of this study were that the web-based integrated management program significantly improved coping strategies, medication adherence, and HRQoL in patients with<br>AF after 1, 3, and 6 months of intervention. In addition, the AF patients receiving the web-based integrated management program had a lower probability of readmission within 2 years after intervention compared with the patients not receiving this program".		
**2a-ii) Scientific background, rationale: What is known about the (type of) system**		
"Prior studies support the use of eHealth to assist patients in conducting advanced disease management. A few randomized controlled trials had investigated the impact of eHealth on medication adherence in patients with AF for 3 months. Although the use of eHealth management significantly improved medication adherence at 3 months after intervention, the long-term effects on medication adherence remain to be investigated. Again, integrated management aims to improve patients' disease knowledge and empower patients to manage their disease. Integrated management needs the involvement of patients and multidisciplinary AF teams. Multidisciplinary professionals using technological support such as eHeath provide disease information and self-care skills to AF patient. As a result,advanced and integrated disease management programs are expected to improve medication adherence and disease control in patients with AF".		
**Does your paper address CONSORT subitem 2b?**		
"In this study, we aimed to evaluate the effects of a web-based integrated management program on improving coping strategies, medication adherence, and health-related quality of life (HRQoL) in patients with AF, and to detect its effect on decreasing readmission events. Therefore, we have two hypotheses. The first hypothesis is that the AF patients receiving<br>the web-based integrated management program in the intervention group would use more approach coping strategies and improve their medication adherence and HRQoL compared<br>with the patients in the control group. The second hypothesis is that the patients in the intervention group would have fewer 2-year readmission events than the patients in the control group".		
METHODS		
**3a) CONSORT: Description of trial design (such as parallel, factorial) including allocation ratio**		
"This study was a prospective, single-blind, randomized controlled trial with repeated measurements to determine the effects of a 6-month web-based integrated management program on improving the coping strategy, medication adherence, and HRQoL, and decreasing 2-year readmission events in patients with AF".<br>"In total, 324 patients were screened in the cardiovascular outpatient department, and 258 patients were eligible to be included. Among the eligible patients, 26 patients refused to<br>participate. The remaining 232 participants were randomly assigned into 2 groups by blocked randomization using a web-based system".		
**3b) CONSORT: Important changes to methods after trial commencement (such as eligibility criteria), with reasons**		
There were no important change to the methods after trial commencement.		
**3b-i) Bug fixes, Downtimes, Content Changes**		
**4a) CONSORT: Eligibility criteria for participants**		
"Patients were included if they met the following criteria: (1) diagnosed with AF by cardiologists, (2) receiving anticoagulant treatment, (3) aged above 20 years, (4)<br>able to speak and read Taiwanese or Mandarin to understand and follow instructions, and (5) able to use a mobile phone or computer correctly. The exclusion criteria included the<br>following: (1) diagnosed with mental disorders or (2) involved in other clinical trials"		
**4a-i) Computer / Internet literacy**		
**4a-ii) Open vs. closed, web-based vs. face-to-face assessments:**		
"Participants were recruited from the cardiovascular outpatient department at a medical center in northern Taiwan through convenience sampling. ...."<br>"The remaining 232 participants were randomly assigned into 2 groups by blocked randomization using a web-based system. Finally, 116 participants were assigned into the intervention group and 116 participants were assigned into the control group...."<br>"The web-based integrated management program was conducted in the cardiovascular outpatient department. Participants had their own account and password to log in to the web-based program via mobile phones or computers........."<br>"The research nurse also sent messages every day to monitor the participants' condition through the messaging function of this domain. When the participants had an emergency event, they could receive help to manage this situation through textual information or telephonic coaching."<br>"After face-to-face presentations, we ensured that the participants fully understood the instructions for using this web-based program by the return demonstration method. We reviewed their understanding of the program at the next outpatient visit. In addition, we instructed participants on managing AF and anticoagulant treatment through the web-based program and provided the AF management manual to them."		
**4a-iii) Information giving during recruitment**		
**4b) CONSORT: Settings and locations where the data were collected**		
Participants were recruited from the cardiovascular outpatient department at a medical center in northern Taiwan through convenience sampling.		
**4b-i) Report if outcomes were (self-)assessed through online questionnaires**		
The research nurse administered self-complete questionnaires to patients at baseline, 1 month, 3 month, and 6 months.		
**4b-ii) Report how institutional affiliations are displayed**		
**5) CONSORT: Describe the interventions for each group with sufficient details to allow replication, including how and when they were actually administered**		
**5-i) Mention names, credential, affiliations of the developers, sponsors, and owners**		
**5-ii) Describe the history/development process**		

**5-iii) Revisions and updating**		
**5-iv) Quality assurance methods**		
**5-v) Ensure replicability by publishing the source code, and/or providing screenshots/screen-capture video, and/or providing flowcharts of the algorithms used**		
**5-vi) Digital preservation**		
**5-vii) Access**		
A secure website was designed to assist patients in performing safe self-management their disease.<br>We assisted participants with free set-up required on their smartphones or tablets.		
**5-viii) Mode of delivery, features/functionalities/components of the intervention and comparator, and the theoretical framework**		
"The program included five domains: patient information collection, instructions on AF knowledge, instructions on anticoagulation medicine, self-monitoring of symptoms, and professional consultation".<br>"In the patient information collection domain, the participants were able to provide and read their information...."<br>"In the instructions on AF knowledge domain, participants were able to receive information about AF through texts and videos..."<br>"In the instructions on anticoagulation medicine domain, the participants were able to obtain descriptions about a variety of anticoagulant medicines...."<br>"In the self-monitoring of symptoms domain, participants could provide record their symptoms every day.."<br>"In the professional consultation domain, participants could receive consultations<br>from multidisciplinary professionals on any issues related to AF at any time..."<br>"All the participants had their own private consultations with clinical professionals."		
**5-ix) Describe use parameters**		
**5-x) Clarify the level of human involvement**		
**5-xi) Report any prompts/reminders used**		
"The research nurse also sent messages every day to monitor the participants' condition through the messaging function of this domain. When the participants had an emergency event, they could receive help to manage this situation through textual information or telephonic coaching (Figures 1 and 2)."		
**5-xii) Describe any co-interventions (incl. training/support)**		
..."a 6-month web-based integrated management program"....<br>"The patients in the intervention group received the web-based integrated management program."		
**6a) CONSORT: Completely defined pre-specified primary and secondary outcome measures, including how and when they were assessed**		
The research nurse administered self-complete questionnaires to patients at baseline, 1 month, 3 month, and 6 months.<br>Primary outcomes:<br>Coping Strategies(Brief Coping Orientation to Problems Experienced scale)<br>Medication Adherence (Medication Adherence Rating Scale)<br>Medication Adherence Rating Scale (three-level version of the EuroQol five-dimension self-report questionnaire (EQ-5D-3L))<br><br>Secondary outcomes:<br>Readmission Events Within 2 Years (After recruiting the patients in the study, we followed their readmission events for 2 years. We collected these data through chart reviews of each participant.)		
**6a-i) Online questionnaires: describe if they were validated for online use and apply CHERRIES items to describe how the questionnaires were designed/deployed**		
**6a-ii) Describe whether and how "use" (including intensity of use/dosage) was defined/measured/monitored**		
**6a-iii) Describe whether, how, and when qualitative feedback from participants was obtained**		
**6b) CONSORT: Any changes to trial outcomes after the trial commenced, with reasons**		
Participants were recruited from the cardiovascular outpatient department at a medical center in northern Taiwan through convenience sampling.		
**7a) CONSORT: How sample size was determined**		
**7a-i) Describe whether and how expected attrition was taken into account when calculating the sample size**		
**7b) CONSORT: When applicable, explanation of any interim analyses and stopping guidelines**		
The research nurse administered self-complete questionnaires to patients at baseline, 1 month, 3 month, and 6 months.<br>Primary outcomes:<br>Coping Strategies(Brief Coping Orientation to Problems Experienced scale)<br>Medication Adherence (Medication Adherence Rating Scale)<br>Medication Adherence Rating Scale (three-level version of the EuroQol five-dimension self-report questionnaire (EQ-5D-3L))<br><br>Secondary outcomes:<br>Readmission Events Within 2 Years (After recruiting the patients in the study, we followed their readmission events for 2 years. We collected these data through chart reviews of each participant.)		
**8a) CONSORT: Method used to generate the random allocation sequence**		
" In total, 324 patients were screened in the cardiovascular outpatient department, and 258 patients were eligible to be included. Among the eligible patients, 26 patients refused to<br>participate. The remaining 232 participants were randomly assigned into 2 groups by blocked randomization using a web-based system....."		
**8b) CONSORT: Type of randomisation; details of any restriction (such as blocking and block size)**		
" In total, 324 patients were screened in the cardiovascular outpatient department, and 258 patients were eligible to be included. Among the eligible patients, 26 patients refused to<br>participate. The remaining 232 participants were randomly assigned into 2 groups by blocked randomization using a web-based system....."		
**9) CONSORT: Mechanism used to implement the random allocation sequence (such as sequentially numbered containers), describing any steps taken to conceal the sequence until interventions were assigned**		
" In total, 324 patients were screened in the cardiovascular outpatient department, and 258 patients were eligible to be included. Among the eligible patients, 26 patients refused to<br>participate. The remaining 232 participants were randomly assigned into 2 groups by blocked randomization using a web-based system....."		
**10) CONSORT: Who generated the random allocation sequence, who enrolled participants, and who assigned participants to interventions**		
The statistician who generated the random allocation sequence by a web-based system. The research nurse who enrolled participants, and assigned participants to intervention based on the allocation sequence.		
**11a) CONSORT: Blinding - If done, who was blinded after assignment to interventions (for example, participants, care providers, those assessing outcomes) and how**		
**11a-i) Specify who was blinded, and who wasn't**		
"A research nurse explained the research process to each participant and obtained informed consent from them."<br>The analysis was conducted by researchers who were unaware of the random allocation.		
**11a-ii) Discuss e.g., whether participants knew which intervention was the "intervention of interest" and which one was the "comparator"**		
**11b) CONSORT: If relevant, description of the similarity of interventions**		
Not applicable to this trial.		
**12a) CONSORT: Statistical methods used to compare groups for primary and secondary outcomes**		
"The effects of the web-based integrated management program on the coping strategy,<br>medication adherence, HRQoL, and readmission events were examined using a generalized estimating equation (GEE). The significance level was defined by a two-tailed t test with<br>P<.05."<br>"We used univariate logistic regression to assess the association between patient characteristics, including group assignment, and readmission events within 2 years and identify the predictor variables. ....."		
**12a-i) Imputation techniques to deal with attrition / missing values**		
Not applicable to this trial.		
**12b) CONSORT: Methods for additional analyses, such as subgroup analyses and adjusted analyses**		

Not applicable to this trial.		
RESULTS		
**13a) CONSORT: For each group, the numbers of participants who were randomly assigned, received intended treatment, and were analysed for the primary outcome**		
"In total, 324 patients were screened in the cardiovascular outpatient department, and 258 patients were eligible to be included. Among the eligible patients, 26 patients refused to<br>participate. The remaining 232 participants were randomly assigned into 2 groups by blocked randomization using a web-based system. Finally, 116 participants were assigned<br>into the intervention group and 116 participants were assigned into the control group. The patients in the intervention group received the web-based integrated management program,<br>whereas those in the control group received consultations and were coached thrice by a research nurse over telephone."<br>"A total of 258 patients were recruited from the outpatient department of the study hospital according to the inclusion criteria. Of these patients, 26 refused to participate. At the end<br>of the study, the complete data of 115 participants in the intervention group and 116 participants in the control group were included for the statistical analysis. One participant in the intervention group was lost to follow-up owing to emigration (Figure 3)."		
**13b) CONSORT: For each group, losses and exclusions after randomisation, together with reasons**		
"A total of 258 patients were recruited from the outpatient department of the study hospital according to the inclusion criteria. Of these patients, 26 refused to participate. At the end<br>of the study, the complete data of 115 participants in the intervention group and 116 participants in the control group were included for the statistical analysis. One participant in the intervention group was lost to follow-up owing to emigration (Figure 3)."		
**13b-i) Attrition diagram**		
**14a) CONSORT: Dates defining the periods of recruitment and follow-up**		
Data were collected from October 2018 to January 2021. We collected the data at 4 time points: baseline (prior to randomization, T0), 1 month after beginning the intervention<br>(T1), after completing 3 months of the intervention (T2), and after completing 6 months of the intervention (T3). As well as, After recruiting the patients in the study, we followed their<br>readmission events for 2 years.		
**14a-i) Indicate if critical "secular events" fell into the study period**		
**14b) CONSORT: Why the trial ended or was stopped (early)**		
Not applicable in this trial. This trial was not stopped early.		
**15) CONSORT: A table showing baseline demographic and clinical characteristics for each group**		
"There were no significant differences in the demographic and clinical characteristics, and the baseline scores of each scale between the two groups (Table 1).		
**15-i) Report demographics associated with digital divide issues**		
"Demographic and clinical characteristics, including age, gender, education, marital status, type of AF, mEHRA classification, comorbidities, and use of anticoagulation medicine, were<br>recorded before random assignment."		
**16a) CONSORT: For each group, number of participants (denominator) included in each analysis and whether the analysis was by original assigned groups**		
**16-i) Report multiple "denominators" and provide definitions**		
The demographic of participants were included age, gender, education, marital status.., as Table 1.		
**16-ii) Primary analysis should be intent-to-treat**		
**17a) CONSORT: For each primary and secondary outcome, results for each group, and the estimated effect size and its precision (such as 95% confidence interval)**		
See Table 2-5 for each primary and secondary outcome.		
**17a-i) Presentation of process outcomes such as metrics of use and intensity of use**		
**17b) CONSORT: For binary outcomes, presentation of both absolute and relative effect sizes is recommended**		
Not applicable to this trial.		
**18) CONSORT: Results of any other analyses performed, including subgroup analyses and adjusted analyses, distinguishing pre-specified from exploratory**		
Not applicable to this trial.		
**18-i) Subgroup analysis of comparing only users**		
**19) CONSORT: All important harms or unintended effects in each group**		
"The web-based integrated management program can significantly improve patients' coping strategies and medication adherence, and help patients maintain disease stability, which has a major influence on improving the HRQoL and reducing adverse clinical events<br>and readmission episodes."<br>Additionally, no harmful events occurred in our trial.		
**19-i) Include privacy breaches, technical problems**		
**19-ii) Include qualitative feedback from participants or observations from staff/researchers**		
DISCUSSION		
**20) CONSORT: Trial limitations, addressing sources of potential bias, imprecision, multiplicity of analyses**		
**20-i) Typical limitations in ehealth trials**		
"Some limitations of this study should be considered. First, the sample may not reflect the entire AF population owing to the disease severity of most patients being mild or moderate. If AF patients had severe symptoms, they were hospitalized to receive medical interventions. Therefore, most of the AF patients in the community only had mild or moderate symptoms. However, they still had very limited knowledge on self-managing their<br>symptoms. After receiving this program, they were able to take care of themselves, leading to decreased readmission events.........."		
**21) CONSORT: Generalisability (external validity, applicability) of the trial findings**		
**21-i) Generalizability to other populations**		
**21-ii) Discuss if there were elements in the RCT that would be different in a routine application setting**		
**22) CONSORT: Interpretation consistent with results, balancing benefits and harms, and considering other relevant evidence**		
**22-i) Restate study questions and summarize the answers suggested by the data, starting with primary outcomes and process outcomes (use)**		
"The main findings of this study were that the web-based integrated management program significantly improved coping strategies, medication adherence, and HRQoL in patients with<br>AF after 1, 3, and 6 months of intervention. In addition, the AF patients receiving the web-based integrated management program had a lower probability of readmission within 2 years after intervention compared with the patients not receiving this program."		
**22-ii) Highlight unanswered new questions, suggest future research**		
Other information		
**23) CONSORT: Registration number and name of trial registry**		
"This study was approved by the Institutional Review Board of the participating hospital (IRB: 2-107-05-081; Clinical Trials.gov, number NCT04813094)"		
**24) CONSORT: Where the full trial protocol can be accessed, if available**		
The full trial protocol can be accessed in this manuscript.		
**25) CONSORT: Sources of funding and other support (such as supply of drugs), role of funders**		
The full trial protocol can be accessed in this manuscript.		
**X26-i) Comment on ethics committee approval**		
**x26-ii) Outline informed consent procedures**		
**X26-iii) Safety and security procedures**		

**X27-i) State the relation of the study team towards the system being evaluated**		
